# The Trithorax Group Factor ULTRAPETALA1 Regulates Developmental as Well as Biotic and Abiotic Stress Response Genes in Arabidopsis

**DOI:** 10.1534/g3.119.400559

**Published:** 2019-10-11

**Authors:** Ludmila Tyler, Mark J. Miller, Jennifer C. Fletcher

**Affiliations:** Plant Gene Expression Center, United States Department of Agriculture-Agricultural Research Service, Albany, California, 94710 and Department of Plant and Microbial Biology, University of California, Berkeley, 94720

**Keywords:** Arabidopsis, microarray, epigenetics, CLF, ULT1

## Abstract

In eukaryotes, Polycomb group (PcG) and trithorax group (trxG) factors oppositely regulate gene transcription during development through histone modifications, with PcG factors repressing and trxG factors activating the expression of their target genes. Although plant trxG factors regulate many developmental and physiological processes, their downstream targets are poorly characterized. Here we use transcriptomics to identify genome-wide targets of the *Arabidopsis thaliana* trxG factor ULTRAPETALA1 (ULT1) during vegetative and reproductive development and compare them with those of the PcG factor CURLY LEAF (CLF). We find that genes involved in development and transcription regulation are over-represented among ULT1 target genes. In addition, stress response genes and defense response genes such as those in glucosinolate metabolic pathways are enriched, revealing a previously unknown role for *ULT1* in controlling biotic and abiotic response pathways. Finally, we show that many ULT1 target genes can be oppositely regulated by CLF, suggesting that ULT1 and CLF may have antagonistic effects on plant growth and development in response to various endogenous and environmental cues.

The spatial and temporal regulation of gene expression is essential for the development of multicellular organisms. In eukaryotes, Polycomb group (PcG) and trithorax group (trxG) proteins control gene transcription and regulate development through the epigenetic modification of chromatin within the nucleus (Schuettengruber *et al.* 2017). PcG factors form complexes that establish and maintain repressive gene expression states, whereas trxG complexes function in various aspects of transcription activation. In plants, as in animals, PcG and trxG factors act in developmental transitions at all major stages of the life cycle and play important roles in cell identity specification and cell fate switches (Mozgova and Hennig 2015, Pu and Sung 2015). The ability of these factors to remodel chromatin and/or modify histones enables them to regulate the expression of thousands of genes; however, which sets of PcG and trxG factors regulate which combinations of target genes in different tissues and at different times during the life cycle remains poorly understood.

PcG factors were originally identified as repressors of *homeobox* (*Hox*) transcription factor genes during *Drosophila melanogaster* embryo development (Lewis 1978). PcG factors form two major complexes in eukaryotes, Polycomb Repressive Complex1 (PRC1) and PRC2, which affect gene silencing through histone modifications to regulate various developmental processes. In Arabidopsis, the CURLY LEAF (CLF) SET domain methyltransferase protein is a core component of PRC2 that tri-methylates lysine 27 of histone H3 (H3K27me3) to repress the transcription of target genes (Schubert *et al.* 2006, Jiang *et al.* 2008). Plants homozygous for loss-of-function *clf* mutations display small rosettes, upward-curling leaves, early flowering and floral organ homeotic transformations, caused by a failure to stably repress flower-specific genes such as the MADS box transcription factor (TF) genes *AGAMOUS* (*AG*) and *APETALA3* (*AP3*) in vegetative tissues (Goodrich *et al.* 1997, Schubert *et al.* 2006). In total, CLF negatively regulates ∼11.6% of Arabidopsis genes in various tissues, with nearly half of these CLF-repressed loci associated with H3K27me3 repressive marks (Liu *et al.* 2016).

trxG factors, which counteract PcG-mediated gene repression, were initially characterized as genetic suppressors of PcG mutant phenotypes. Multiple Arabidopsis trxG genes have been identified either on this basis or through their homology to animal trxG genes. Yet compared to PcG factors, trxG factors are poorly characterized in plants. Plant trxG factors fall into two broad functional categories, ATP-dependent chromatin remodeling proteins and histone-modifying proteins (Mozgova and Hennig 2015). Those in the latter category display H3K4 and/or H3K36 methyltransferase activity. Arabidopsis contains a family of H3K4me3 methyltransferase genes encoding SET domain proteins with homology to Drosophila Trithorax (Alvarez-Venegas and Avramova 2001, Baumbusch *et al.* 2001). Among these family members, ARABIDOPSIS HOMOLOG OF TRITHORAX1 (ATX1) is a component of the AtCOMPASS complex (Jiang *et al.* 2009, Jiang *et al.* 2011) that is important for recruiting RNA Polymerase II to its target gene promoters (Ding *et al.* 2011a) and for H3K4me3 deposition associated with transcription elongation (Ding *et al.* 2012b). ATX1 deposits ∼15% of H3K4me3 in the genome (Alvarez-Venegas and Avramova 2005) and has pleiotropic effects on Arabidopsis development (Alvarez-Venegas *et al.* 2003, Pien *et al.* 2008, Napsucialy-Mendivil *et al.* 2014), as well as on biotic and abiotic stress responses (Alvarez-Venegas *et al.* 2006, Ding *et al.* 2011b).

The SAND domain protein ULTRAPETALA1 (ULT1) functions as a trxG factor and physically associates with ATX1 (Carles and Fletcher 2009). *ULT1* and the paralogous *ULT2* gene function during development to regulate shoot and floral meristem activity and to pattern the gynoecium (Fletcher 2001, Monfared *et al.* 2013, Pires *et al.* 2014). Although the ULT proteins lack sequence homology with known animal trxG factors, *ult1* loss-of-function alleles fully suppress the *clf* null mutant phenotypes, and ULT1 limits the ability of CLF to deposit H3K27me3 at target gene loci such as *AG* and *AP3*, thus acting as a PcG anti-repressor (Carles and Fletcher 2009). Eliminating *ULT1* function also rescues the severe vegetative and floral development defects of *LFYasEMF1* transgenic plants in which the PcG gene *EMBRYONIC FLOWER1* (*EMF1*) is down-regulated shortly after germination (Pu *et al.* 2013). *ult1* mutations restore the proper expression levels of many classes of genes mis-regulated in *LFYasEMF1* plants; accordingly, reducing *ULT1* activity increases H3K27me3 repressive marks and decreases H3K4me3 active marks at these target genes. Removing both EMF1 and ULT1 activities restores the two types of methylation marks to near wild-type levels, indicating that ULT1 counteracts both CLF and EMF1 action during vegetative and floral development via modulation of histone marks on a wide variety of target genes. However, unlike other Arabidopsis trxG genes, which are broadly expressed, *ULT1* and *ULT2* transcription occurs predominantly in meristems and young organ primordia throughout development (Carles *et al.* 2005), suggesting the *ULT* genes may function in a more tissue-restricted fashion than other plant trxG genes.

In addition to its interaction with ATX1, ULT1 physically associates with several sequence-specific DNA-binding transcription factors. These include the Myb domain transcription factor ULTRAPETALA INTERACTING FACTOR1 (UIF1) (Moreau *et al.* 2016) and, through its interaction with the ULT2 protein, the GARP domain transcription factors KANADI1 (KAN1) and KAN2 (Pires *et al.* 2014). These associations suggest that the ULT proteins may physically link sequence-specific TFs with histone methyltransferases and the transcription machinery. The UIF1, KAN1 and KAN2 TFs all bind to functional Polycomb response elements (Xiao *et al.* 2017), and the rice OsULT1 protein itself directly binds a “GAGAG” motif present in Polycomb response elements (Roy *et al.* 2019). Therefore the ULT proteins may play an important function in coordinating the specific placement of histone-modifying enzymes at target gene loci.

Although ULT1 can counteract CLF function, it is not known how broadly ULT1 regulates gene transcription during normal Arabidopsis development, nor whether ULT1 opposes CLF action on a wide scale or at only a few key target loci. Here we analyze the ULT1 transcriptome at the vegetative and reproductive stages using both loss- and gain-of-function lines, and compare it directly with the CLF transcriptome. We show that *ULT1* regulates only 2.6% of Arabidopsis genes during development, far fewer than other Arabidopsis trxG factors and consistent with a role in a subset of chromatin-associated activities. Genes involved in plant development and transcription are over-represented among ULT1-regulated genes, as are stress-responsive genes and immune response genes such as those in the glucosinolate biosynthesis and breakdown pathways. These data reveal a previously unknown role for ULT1 in controlling biotic and abiotic responses. Finally we demonstrate that many CLF target genes can be oppositely regulated by ULT1, indicating that ULT1 may have a broad function in opposing PRC2-mediated transcription repression during Arabidopsis growth and development.

## Materials and Methods

### Plant materials

All *Arabidopsis thaliana* lines are in the Landsberg *erecta* (L*er*) background and have been previously described (Goodrich *et al.* 1997, Carles *et al.* 2005, Carles and Fletcher 2009). Arabidopsis seeds were sown either on Murashige and Skoog plates or in soil (50% medium vermiculite and 50% Sunshine Mix #1) and stratified for 5 days at 4° before being transferred to a growth chamber under constant light conditions (∼120 μmol m^-2^ s^-1^ light intensity) at 21°. Following germination the plants in soil were fertilized daily with a dilute mixture of Miracle Grow 20-20-20 fertilizer.

### Microarray and gene ontogeny analysis

For the vegetative stage analysis, shoot apices were collected from 4-day-old seedlings after removal of the cotyledons and roots. For the reproductive stage analysis, inflorescence meristems (IFMs) with unopened flower buds were collected when the stems reached 1 cm in height. Tissue collected from at least 20 randomly chosen plants of each genotype and stage was pooled and immediately flash-frozen in liquid nitrogen, then stored at -80° until RNA extraction. RNA extraction was performed using an RNeasy Plant Mini Kit (Qiagen). For each genotype and stage, samples from three independent biological replicates were hybridized in triplicate (for three technical replicates) to Arabidopsis ATH1 Whole Genome Array Gene Chips (Affymetrix). Raw gene expression data were analyzed using the Bioconductor microarray analysis package (Huber *et al.* 2015), with the Limma empirical Bayes analysis pipeline (Ritchie *et al.* 2015) set at default settings used to detect differentially expressed probes. Cutoff criteria for differential gene expression between samples were a minimum fold-change of 1.5 and an adjusted *p*-value lower than 0.05. Gene ontology (GO) term enrichment analysis was performed using the agriGO v2 online platform Singular Enrichment Analysis (SEA) tool (Du *et al.* 2010), with enrichment calculated relative to the ATH1 Whole Genome Array gene reference list using a hypergeometric test followed by Benjamin-Yekutieli false discovery rate (FDR) correction. GO enrichment analysis was performed using the complete list of plant GO categories; however, the plant GO slim gene ontology analysis option was used to generate most of the hierarchical tree graphs (Figure S2-S8) to reduce the volume of GO sub-categories returned. Venn diagrams were generated using the Venny 2.0 interactive online tool.

### Quantitative RT-PCR

Total RNA was isolated from IFM plus flower bud tissue using an RNeasy Plant Mini Kit. RNA was converted into cDNA using an iScript Reverse Transcription Supermix (Bio-Rad), and quantitative RT-PCR was performed with an iTaq Universal SYBR Green Supermix (Bio-Rad). PCR reactions were run and analyzed using a CFX96 Real-Time PCR Detection System (Bio-Rad). Two-step PCR conditions were as follows: initial denaturation at 95° for 3 min, followed by 40 cycles of 95° for 10 sec and either 57° or 60° for 30 sec. Quantification of relative gene expression was performed using the ∆∆Ct method (Livak and Schmittgen 2001) and calculated from three biological replicates with three technical replicates each. Relative mRNA expression levels were normalized to the *TUBULIN2* (*TUB2*) reference gene and expressed as a ratio to the level in wild-type plants. Primers are listed in Table S8.

### Data availability

The microarray raw data generated in this study are available from the NCBI GEO database under accession number GSE137976. Supplemental material consisting of 8 supplemental tables and 8 supplemental figures are available at figshare: https://doi.org/10.25387/g3.8967986.

## Results

### Genome-wide expression analyses of ULT1 target genes

**Figure 1:**
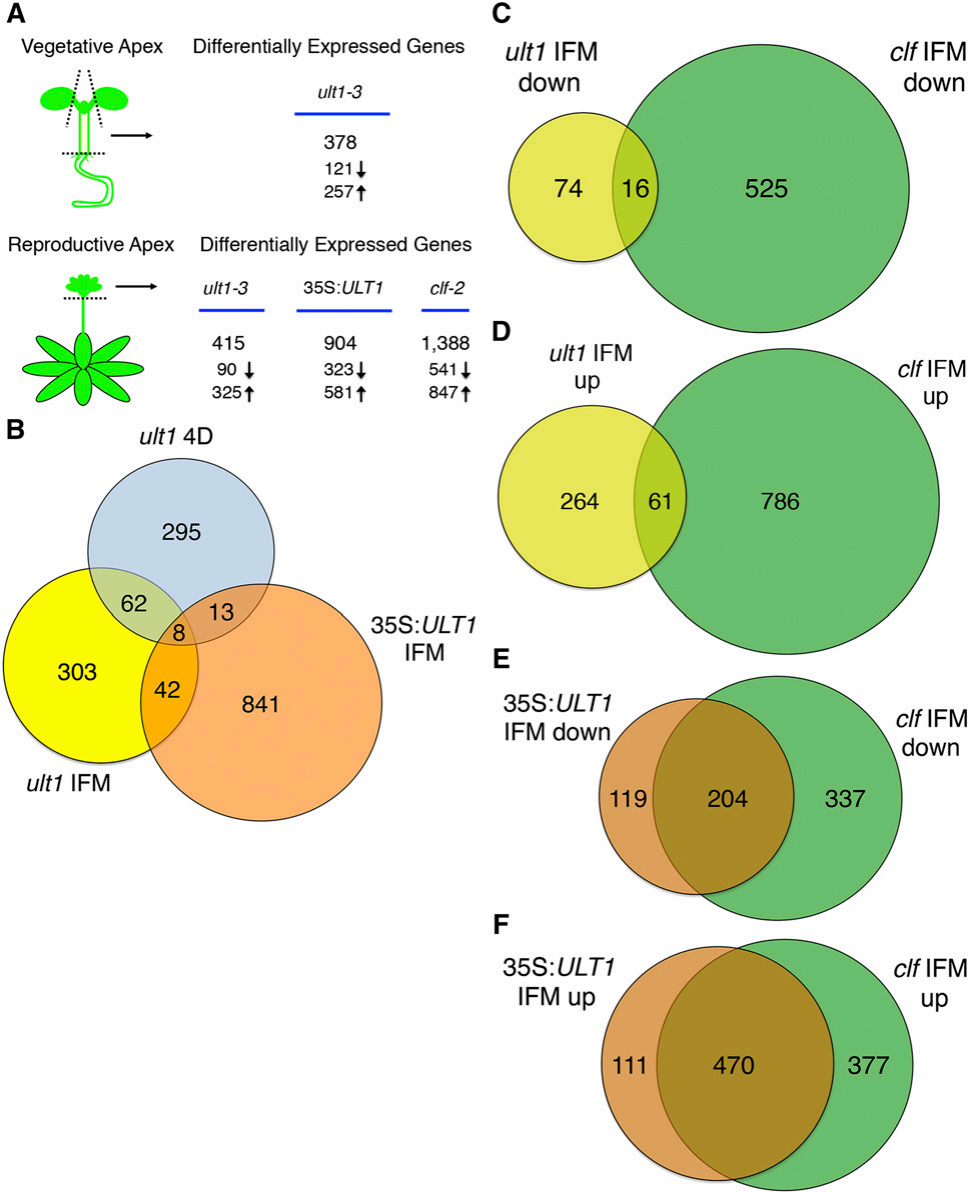
Microarray analysis of *ULT1* and *CLF* target genes. A. Schematic of enrichment for meristematic tissues and number of differentially expressed genes (DEGs) (*P* ≤ 0.05 and FC ≥1.5) compared to wild-type for each genotype at the vegetative (top) or reproductive (bottom) stage. Down arrow indicates down-regulated genes and up arrow indicates up-regulated genes. B. Venn diagram showing overlap between DEGs in *ult1-3* 4-day-old (4D) vegetative apices, *ult1-3* IFM apices, and 35S:*ULT1* IFM apices. Genes were considered to overlap if they displayed the same direction of fold change (FC) in *ult1-3* vegetative and IFM apices, or the opposite direction of FC in 35S:*ULT1* IFM apices and *ult1-3* vegetative and/or IFM apices. C. Venn diagram showing overlap between down-regulated DEGs in *ult1-3* and *clf-2* IFM apices. D. Venn diagram showing overlap between up-regulated DEGs in *ult1-3* and *clf-2* IFM apices. E. Venn diagram showing overlap between DEGs displaying the same direction of FC that are down-regulated in both 35S:*ULT1* IFM apices and *clf-2* IFM apices. F. Venn diagram showing overlap between DEGs displaying the same direction of FC that are up-regulated in both 35S:*ULT1* IFM apices and *clf-2* IFM apices.

To identify *ULT1*-regulated genes during Arabidopsis development, we performed whole-genome transcription profiling of wild-type L*er*, *ult1-3*, 35S:*ULT1* and *clf-2* plants. The *ult1-3* allele is a T-DNA null allele originally identified in the Col-0 background (Carles *et al.* 2005) that was introgressed five times into the L*er* background prior to analysis. The 35S:*ULT1* transgenic plants are in the L*er* background and display a strong gain-of-function phenotype of curled leaves, small rosettes, premature flowering and floral homeotic transformations (Carles and Fletcher 2009) strikingly similar to that of plants homozygous for the *clf-2* Ds null allele in the L*er* background (Goodrich *et al.* 1997). Each genotype was analyzed at the reproductive stage when the main stem reached 1 cm in height. Because *ULT1* is preferentially expressed in shoot and floral meristems (Carles *et al.* 2005), we enriched for meristematic tissues by collecting inflorescence meristem (IFM) apices with unopened flower buds from reproductive-stage plants (Figure 1A). We also collected vegetative shoot apices minus roots and cotyledons from seedlings 4 days after germination (4 DAG); however, because 35S:*ULT1* and *clf-2* seedlings are tiny and undergo the transition to flowering very prematurely under constant light conditions, we were unable to collect sufficient vegetative tissue from these two genotypes for robust analysis.

Affymetrix gene chips (ATH1 Whole Genome Arrays) representing ∼24,000 Arabidopsis genes were used in the whole-genome expression analysis. Correlation coefficients close to 1.0 for all samples tested indicated the high reproducibility of the experiments (Table S1). Genes with a ≥1.5-fold expression change and a *p*-value ≤0.05 compared to the wild-type were considered to have significantly different expression levels. Differentially expressed genes (DEGs) identified in the mutant and transgenic plants are listed in Supplementary Tables S2-5. Because transcriptomics analysis does not distinguish between primary and secondary effects on gene transcription, these DEGs represent both direct and indirect targets of ULT1 and CLF.

A total of 378 genes were differentially expressed in *ult1-3* 4 DAG seedling apices compared to wild-type apices (Figure 1A, Table S2), representing approximately 1.6% of the total number of Arabidopsis genes sampled. Among these, 121 genes were down-regulated and 257 were up-regulated. A total of 415 genes were differentially expressed in *ult1-3* IFM apices compared to wild-type apices (Table S3). Among these, 90 genes were down-regulated and 325 were up-regulated. Over twice as many genes, a total of 904, were differentially expressed in 35S:*ULT1* IFM apices compared to wild-type apices (Table S4), representing 3.8% of the total genes sampled. Among these, 323 genes were down-regulated and 581 were up-regulated. Therefore, ULT1 activity leads directly or indirectly to both activation and repression of downstream gene transcription.

We found a total of 723 genes were regulated by ULT1 in vegetative and/or IFM apices (Figure 1B). This corresponds to 3.0% of the genes represented on the microarray or 2.6% of the 27,655 protein-coding loci in the Arabidopsis genome (Cheng *et al.* 2017). Among these 723 genes, 70 were regulated by ULT1 in vegetative and IFM apices with the same direction of fold change (FC) in both samples, 24 of which were down-regulated and 46 of which were up-regulated in *ult1-3* plants. In contrast, 308 genes were ULT1-regulated specifically in vegetative apices and 345 genes specifically in IFM apices (Figure 1B). These data suggest that ULT1 largely regulates gene expression in shoot apex tissues in a stage-specific fashion during development. The data also reveal that ULT1 regulates a relatively small number of genes compared to other known Arabidopsis trxG and PcG factors, although our enrichment for meristematic tissues means that our dataset may under-represent the total number of ULT1-regulated genes in whole plants.

Analysis of the CLF transcriptome revealed a total of 1,388 differentially expressed genes in *clf-2* IFM apices compared to wild-type apices (Figure 1A), representing 5.8% of the genes sampled and 5% of the Arabidopsis genome overall. Among these, 541 were down-regulated and 847 were up-regulated (Table S5). When the 1,388 DEGs in *clf-2* IFM apices were compared to the 415 DEGs in *ult1-3* IFM apices, a total of 128 genes were regulated by both proteins. Thus 30.8% of the ULT1-regulated genes in IFM apices are also regulated by CLF. Within this dataset 16 DEGs were induced by both ULT1 and CLF (Figure 1C) and 61 DEGs were repressed (Figure 1D), indicating cooperative regulation of 77 genes, whereas the other 51 genes were oppositely regulated by ULT1 and CLF. When the DEGs from *clf-2* IFM apices were compared to the 904 DEGs in 35S:*ULT1* IFM apices, a total of 674 genes with the same direction of FC were shared. Within this dataset, 204 DEGs were by repressed by ULT1 and induced by CLF (Figure 1E), and 470 DEGs were induced by ULT1 and repressed by CLF (Figure 1F). Therefore 74.6% of genes that are mis-regulated in *ULT1*-over-expressing lines are oppositely regulated by the PcG factor CLF. This finding indicates that *ULT1*-over-expressing plants resemble *clf-2* plants in their global transcription profiles as well as in their macroscopic phenotypes and is consistent with trxG factors and PcG factors acting antagonistically on target gene expression.

### Functional categorization of ULT1 and CLF target genes

Because differences in gene expression underlie different biological functions, we used gene ontology (GO) term enrichment analysis to elucidate the functions of the differentially expressed genes. We utilized the agriGO web application (Du *et al.* 2010) to assess the over-representation of GO categories in networks of biological processes for down-regulated and up-regulated genes among the different genotypes and tissue types. The resulting GO distribution datasets were visualized as hierarchical tree graphs using Singular Enrichment Analysis (SEA), with enrichment calculated relative to the ATH1 Whole Genome Array gene reference list using a hypergeometric test followed by Benjamin-Yekutieli false discovery rate (FDR) correction. GO terms with adjusted *p*-values less than 0.05 were considered to be significantly over-represented.

The resulting GO distribution networks clearly distinguish between the down-regulated and up-regulated genes in *ult1-3* 4 DAG vegetative apices. The most significant GO terms over-represented among the 121 down-regulated genes fall into several main categories: *response to stimulus*, *metabolic process* and *regulation of biological quality* (Figure S1). Within the *response to stimulus* category, genes categorized as responding to abiotic stimulus, endogenous (hormone) stimulus, chemical stimulus and stress stimulus are significantly enriched. The GO term *oxidation/reduction* within the *metabolic process* category is the most-significantly over-represented (*P* < 7.72e-21) among the down-regulated genes, whereas terms related to cellular iron ion homeostasis appear within the *regulation of biological quality* category. These terms suggest a role for *ULT1* in seedlings to induce the expression of genes involved in abiotic stress responses, redox reactions and cellular ion homeostasis. The main GO terms over-represented among the 257 up-regulated genes grouped into top-level categories comprising *developmental process*, *metabolic process*, *biological regulation*, *multi-organism process* and *response to stimulus* (Figure S2). Within the *developmental process* GO category, genes involved in root development are significantly enriched, whereas within the *metabolic process* GO category, genes associated with oxidation/reduction, glycoside metabolic processes, transcription and protein modification processes are over-represented. The *multi-organism process* and *response to stimulus* GO categories converge on sub-categories of genes associated with innate and induced defense responses, and responses to endogenous (hormone) stimulus. These terms suggest that *ULT1* functions during the vegetative phase to repress biotic stress responses such as innate immune responses and induced defense responses. The results also reveal a potential role for ULT1 to repress aspects of root development in seedling apices.

**Figure 2:**
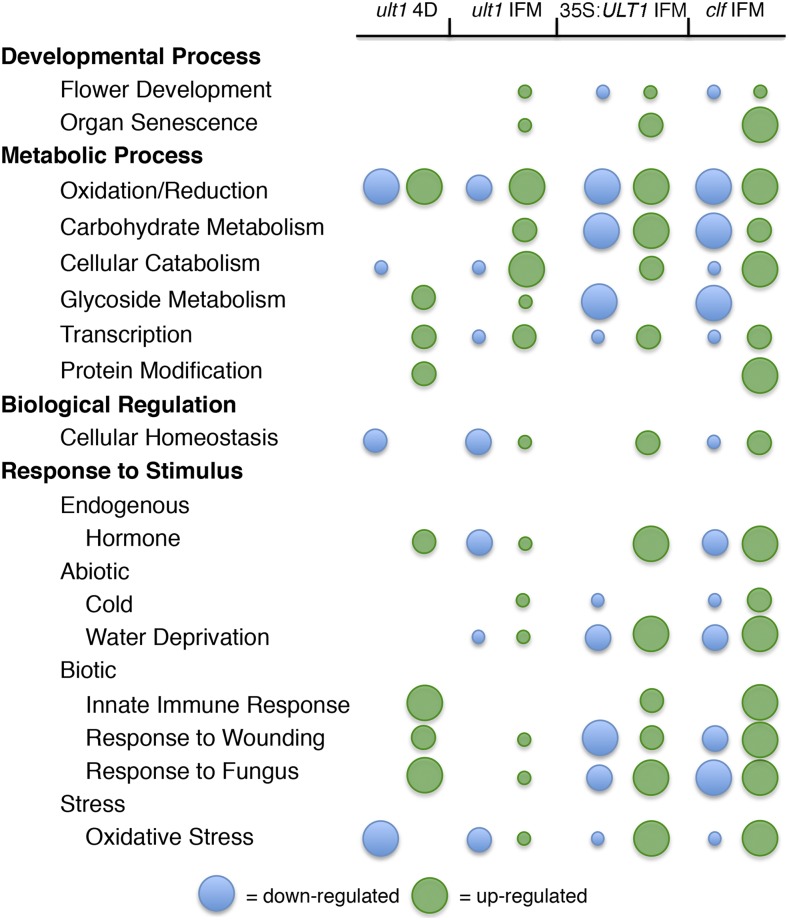
Convergence of significantly enriched GO terms among differentially expressed genes in *ult1-3* 4-day-old (4D) vegetative apices, *ult1-3* IFM apices, 35S:*ULT1* IFM apices and/or *clf-2* IFM apices. Blue color represents down-regulated genes, and green color represents up-regulated genes. Increasing circle size is positively correlated with an increasingly significant *p*-value of the enrichment of the GO term, with small size *P* < 0.05, medium size *P* < 5e-5, and large size *P* < 5e-10.

The GO distribution networks of down-regulated and up-regulated genes in *ult1-3* IFM apices also show clear delineation. The main GO categories over-represented among the 90 down-regulated genes are *response to stimulus* and *biological regulation* (Figure S3). Within the *response to stimulus* category, genes involved in responses to hormone stimulus, water deprivation and oxidative stress stimulus are significantly enriched. Within the *biological regulation* category, genes associated with cellular homeostasis are significantly enriched, as are those involved in oxidation/reduction, cellular catabolic processes and transcription. The majority of these terms are also over-represented among the genes down-regulated in *ult1* seedlings (Figure 2), indicating that ULT1 induces the expression of many of the same classes of genes during vegetative and reproductive growth.

The major over-represented GO categories among the 325 up-regulated genes in *ult1-3* IFM apices comprise *reproduction*, *developmental process*, *metabolic process*, *multi-organism process* and *response to stimulus* (Figure S4). The first two terms converge on the sub-categories of *reproductive development* and *organ senescence*. Within the *metabolic process* category, genes involved in carbohydrate metabolic processes, cellular catabolism, oxidation/reduction, glycoside metabolic processes and transcription are over-represented. The *multi-organism process* and *response to stimulus* terms converge on the sub-categories of endogenous hormone responses and stress responses, as well as responses to both biotic and abiotic stimuli. Genes involved in oxidation/reduction, glycoside metabolic processes, transcription, hormone responses, wounding responses and defense responses are also over-represented among the genes up-regulated in *ult1* seedlings (Figure 2), indicating that *ULT1* represses these classes of genes during both the vegetative and reproductive phases. However, ULT1 appears to regulate developmental gene expression in a stage-specific manner, repressing genes associated with root development in seedlings but those associated with reproductive development in inflorescences.

Differentially expressed genes in 35S:*ULT1* IFM apices fall into main GO categories similar to those in *ult1* IFM apices (Figure S5). However, because these plants over-express *ULT1*, some GO categories may reflect enrichment for genes that are not regulated by *ULT1* under normal physiological conditions. To identify potential biologically relevant target genes during the reproductive phase, we compared the GO terms over-represented among the 323 down-regulated genes in 35S:*ULT1* IFM apices with those of the genes up-regulated in *ult1-3* IFM apices (Figure S4). We found that within the *reproduction* and *developmental process* categories, genes involved in flower development are shared, whereas in the *metabolic process* category, GO terms for genes associated with oxidation/reduction, carbohydrate metabolic processes, glycoside metabolic processes and transcription are shared (Figure 2). The *multi-organism process* and *response to stimulus* categories share sub-categories such as abiotic stress responses to water deprivation and cold, chemical response to oxidative stress, and biotic stress responses to wounding and to fungus. These GO terms are thus likely to represent categories of *bona fide* target genes repressed by ULT1 in IFM apices.

We performed a similar comparison between the GO categories over-represented among the 581 up-regulated genes in 35S:*ULT1* IFM apices (Figure S6) and those of *ult1-3* down-regulated genes (Figure S3). Within the *metabolic process* category, GO terms for genes associated with oxidation/reduction, cellular catabolic processes and transcription are shared, whereas within the *biological regulation* category, the sub-category of cellular homeostasis is shared (Figure 2). The *response to stimulus* category contains the shared GO terms representing genes involved in responses to oxidative stress and water deprivation, as well as those in hormone-mediated signaling pathways. These enriched GO categories represent classes of genes that are likely to be induced by ULT1 during the reproductive phase.

Last, we analyzed the GO distribution networks of DEGs from *clf-2* IFM apices. Within the major GO categories over-represented among the 541 down-regulated genes (Figure S7), the *reproduction* and *developmental process* terms converge on genes with roles in flower, pollen gamete, and seed development, whereas the *biological regulation* category contains genes associated with cell differentiation and cell growth (Figure 2). For the *metabolic process* category, genes involved in various metabolic processes, including glycosides, as well as in cellular biosynthetic processes, oxidation/reduction, and transcription are over-represented. In the *response to stimulus* category genes categorized as responding to abiotic stimuli, such as cold and water deprivation, and biotic stimuli are significantly enriched, along with genes involved in hormone signaling pathways. Within the top-level GO categories enriched among the 847 up-regulated genes (Figure S8), the *reproduction* and *developmental process* terms converge on genes involved in flower development, organ senescence and cell death. The *biological regulation* category contains genes with roles in cellular homeostasis and signal transduction. The *metabolic process* sub-categories are enriched for genes involved in the regulation of various metabolic processes, oxidation/reduction, transcription, and protein modification. Within the *response to stimulus* category, genes categorized as responding to biotic stimuli including innate immune as well as defense responses are significantly enriched, along with abiotic stress responses. Finally, genes involved in several hormone signaling pathways are also over-represented among biological processes repressed by CLF activity during the inflorescence phase.

### Identification of transcription factor genes among DEGs

Genes involved in the regulation of transcription are enriched in each DEG list from our microarray dataset except for those genes down-regulated in *ult1-3* 4 DAG vegetative apices. This GO category includes genes encoding canonical transcription factors (TFs) as well as transcriptional co-regulators and regulatory co-factors. The over-representation of transcriptional regulatory genes indicates that both ULT1 and CLF play important roles in transcriptome modulation.

**Figure 3:**
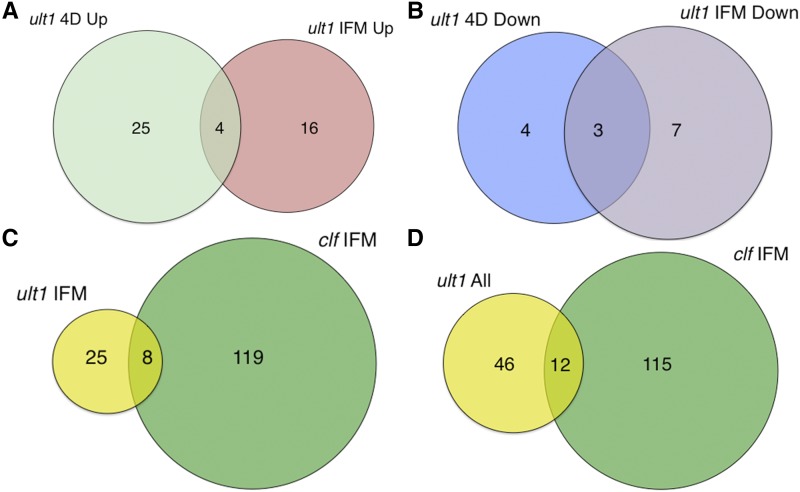
Differentially expressed transcription factor (TF) genes. A. Venn diagram showing overlap between up-regulated TF genes in *ult1-3* 4D vegetative apices and IFM apices. B. Venn diagram showing overlap between down-regulated TF genes in *ult1-3* 4D vegetative apices and IFM apices. C. Venn diagram showing overlap between all differentially expressed (up- or down-regulated) TF genes in *ult1-3* and *clf-2* IFM apices. D. Venn diagram showing overlap between all differentially expressed TF genes in *ult1-3* 4D vegetative apices and IFMs and in *clf-2* IFM apices. Up = up-regulated; Down = down-regulated.

Based on the transcriptome data, ULT1 appears to function predominantly as a repressor of transcriptional regulatory gene expression (Table 1, Table S7). In vegetative apices, the TF families with the largest numbers of genes repressed by ULT1 are the AP2/ERF (6), WRKY (4), and MYB (3) families. AP2/ERF TFs are regulators of abiotic stress responses (Xie *et al.* 2019), whereas WRKY TFs modulate both abiotic and biotic stress responses (Phukan *et al.* 2016) and MYB TFs regulate stress responses as well as metabolism and development (Dubos *et al.* 2010). Among the ULT1-repressed TF genes in reproductive apices, the most heavily represented TF genes are members of the CCAAT and MADS families, which have well-documented roles in development (Smaczniak *et al.* 2012, Zhao *et al.* 2017). Among the transcriptional regulatory genes repressed by ULT1, only four (8.9%) are repressed by ULT1 during both the vegetative and reproductive stages (Figure 3A): the MADS box gene *FLC*, the AP2/ERF gene *RELATED TO AP2.3* (*RAP2.3*), the AUX/IAA gene *INDOLE-3-ACETIC ACID7* (*IAA7*), and the *HISTONE ACETYLTRANSFERASE OF THE CBP FAMILY 1* (*HAC1*) gene, which encodes a transcription co-activator with histone acetyltransferase activity (Pandey *et al.* 2002). Therefore ULT1 largely regulates different sets of transcription factor genes at different stages of development.

**Table 1:** ULT1-regulated transcription factor genes

TF Family	Subfamily	TF Locus ID	Gene Name	logFC	P-value
**ULT1 Repressed 4 DAG**					
AP2/ERF	ERF	At3g15210	ERF4, RAP2.5	0.66	4.10E-04
		At5g47230	ERF5, MACD1	1.34	0.00101
		At1g19210	ERF17	1.63	5.70E-04
		At3g16770	ERF72, ATEPB, RAP2.3	2.13	1.09E-06
		At4g34410	ERF109, RRTF1	2.88	7.94E-04
	RAV	At1g68840	RAV2, TEM2	1.36	7.41E-05
WRKY		At2g47260	WRKY23	0.73	6.10E-04
		At1g80840	WRKY40	1.88	1.11E-04
		At5g49520	WRKY48	1.02	2.11E-04
		At2g25000	WRKY60	0.68	8.14E-04
MYB		At2g16720	MYB7	0.62	3.75E-04
		At1g18570	MYB51, HIG1	1.54	1.29E-04
		At3g50060	MYB77	0.90	6.42E-04
Homeobox	HD-Zip I	At3g01220	ATHB20	1.09	6.68E-06
	BEL	At1g19700	BEL10	0.65	0.000809994
AT-hook		At4g14465	AHL20	0.89	3.69E-04
bZIP		At1g06850	bZIP52	0.88	1.83E-04
bHLH		At3g19860	bHLH121	1.01	1.17E-04
C2H2		At1g27730	STZ, ZAT10	1.67	7.16E-04
DOF		At1g69570	CDF5	0.86	5.95E-05
HSF		At3g24520	HSFC1	1.35	0.00101
LBD		At2g42430	LBD16	1.03	1.03E-04
MADS		At5g10140	FLC	1.65	1.00E-06
NAC		At5g63790	ANAC102	0.70	0.00050
**ULT1 Repressed IFM**					
CCAAT		At3g05690	NF-YA2, HAP2B, UNE8	0.82	4.00E-05
		At1g72830	NF-YA3, HAP2C	0.75	4.00E-05
		At1g54160	NF-YA5	0.68	6.28E-06
		At5g06510	NF-YA10	0.89	0.00011
MADS		At1g26310	AGL10, CAL	1.28	7.58E-07
		At1g22590	AGL87	0.81	2.49E-07
		At5g10140	FLC	2.44	2.59E-09
AP2/ERF	ERF	At3g16770	ERF72, ATEPB, RAP2.3	2.74	5.94E-08
bZIP		At1g35490		0.69	4.60E-04
C2H2-YAB		At1g69180	CRC	0.68	9.76E-07
MYB		At1g66390	MYB90, PAP2	0.82	5.31E-06
NAC		At3g04070	ANAC47, SHG, SHYG	0.60	0.00085
ZF B-box		At3g21890	BBX31, MIP1A	0.61	5.50E-04
**ULT1 Induced 4 DAG**					
bHLH		At5g46760	MYC3	−1.36	2.84E-06
CCAAT		At1g17590	NF-YA8	−0.67	0.000507287
MADS		At2g45660	AGL20, SOC1	−0.84	0.000179044
PHD		At3g24010	ATING1, ING1	−1.02	1.05E-05
ZF		At1g32540	LOL1	−0.60	0.000328726
**ULT1 Induced IFM**					
AP2/ERF	ERF	At5g61600	ERF104	−0.73	1.44E-04
B3	RAV	At2g36080	ABS2, NGAL1	−0.72	3.73E-04
bHLH		At5g46760	MYC3	−1.47	5.81E-09
PHD		At3g24010	ATING1, ING1	−0.83	3.02E-06
TAZ		At3g48360	BT2	−1.18	3.00E-06

The small group of ULT1-induced TF genes is distributed among different families, all of which play roles in plant development or defense responses (Table 1, Table S7). The B3 gene *ABNORMAL SHOOT2* (*ABS2*) and the *ENHANCER OF TRANSCRIPTION1 (ET1)* DNA- and zinc-binding protein gene have pleiotropic growth effects during vegetative and reproductive development (Shao *et al.* 2012, Tedeschi *et al.* 2018), whereas the *SUPPRESSOR OF OVEREXPRESSION OF CONSTANS1* (*SOC1*) and *BTB AND TAZ DOMAIN2* (*BT2*) genes have more restricted developmental activities (Moon *et al.* 2003, Robert *et al.* 2009). The basic helix-loop-helix (bHLH) TF gene *MYC3* controls multiple aspects of jasmonate-mediated plant development and defense responses (Fernández-Calvo *et al.* 2011, Schweizer *et al.* 2013), whereas *ETHYLENE RESPONSE FACTOR104* (*ERF104*) plays a role in pathogen resistance downstream of ethylene signaling (Bethke *et al.* 2009). Three transcriptional regulatory genes are induced by ULT1 during both vegetative and reproductive growth (Figure 3B), *MYC3* as well as *ARABIDOPSIS THALIANA INHIBITOR OF GROWTH1* (*ATING1*) and a high mobility group (HMG1/2) family gene. Induction of these TF genes by ULT1 reflects its known function in regulating developmental processes and is consistent with an additional role in plant defense responses.

CLF also appears to primarily act as a repressor of transcription factor gene expression. CLF represses 86 TF genes in IFM apices, the most highly represented of which are members of the WRKY (13), NAC (9), MADS (5) and MYB (5) gene families (Table S7). In addition to those TF families mentioned above, NAC TFs function in stress responses and in development (Shao *et al.* 2015). In contrast, the 40 CLF-induced TF genes are a much more heterogeneous population that contains six MYB TF genes and three GATA TF genes as well as representatives from more than a dozen other TF families. The common functional themes associated with these CLF-induced TF genes are those of development, represented by genes such as *AUXIN RESPONSE FACTOR4* (*ARF4*), *SHORT VEGETATIVE PHASE* (*SVP*) and *CUP-SHAPED COTYLEDON3* (*CUC3*), and stress responses, represented by genes such as *DREB1A*, *DREB2F* and *ADAP*.

Only a small number of TF and transcription-related genes are regulated by both ULT1 and CLF. Within reproductive apices, only 5.3% of the total ULT1-regulated and CLF-regulated TF genes are shared (Figure 3C). Among these are the MADS TF genes *FLC* and *AGL87*, the NAC TF genes *ANAC047* and *ANAC102*, and the bHLH TF gene *MYC3*. When ULT1-regulated TFs from the vegetative phase are included, a total of 6.9% are shared (Figure 3D). These data suggest that the antagonistic effects of ULT1 and CLF on plant development and target gene transcription occur not entirely through opposite regulation of a small set of key TF genes sitting atop gene regulatory hierarchies, but also through the modulation of genes acting downstream at various points within the molecular pathways.

### Identification of glycoside metabolic process genes among DEGs

Our microarray data indicate that genes involved in glycoside metabolic processes, particularly glycosinolate and glucosinolate (GSL) biosynthetic and metabolic processes, are regulated by ULT1 and CLF (Tables S2-S5). GSLs are sulfur-rich secondary metabolites whose breakdown products play prominent roles in plant-pathogen and plant-herbivore interactions (Wittstock and Burow 2010). GSLs are classified as aliphatic, aromatic, or indolic depending on their amino acid precursor. Biosynthesis occurs through chain elongation of the amino acid precursor, formation of the core GSL structure, and secondary modification of the amino acid side chain (Sønderby *et al.* 2010). GSLs themselves are not bioactive, but are hydrolyzed into toxic breakdown compounds as part of the plant defense response (Wittstock *et al.* 2003). The regulatory networks that control GSL accumulation include metabolic networks as well as biotic and abiotic signaling cascades.

**Figure 4:**
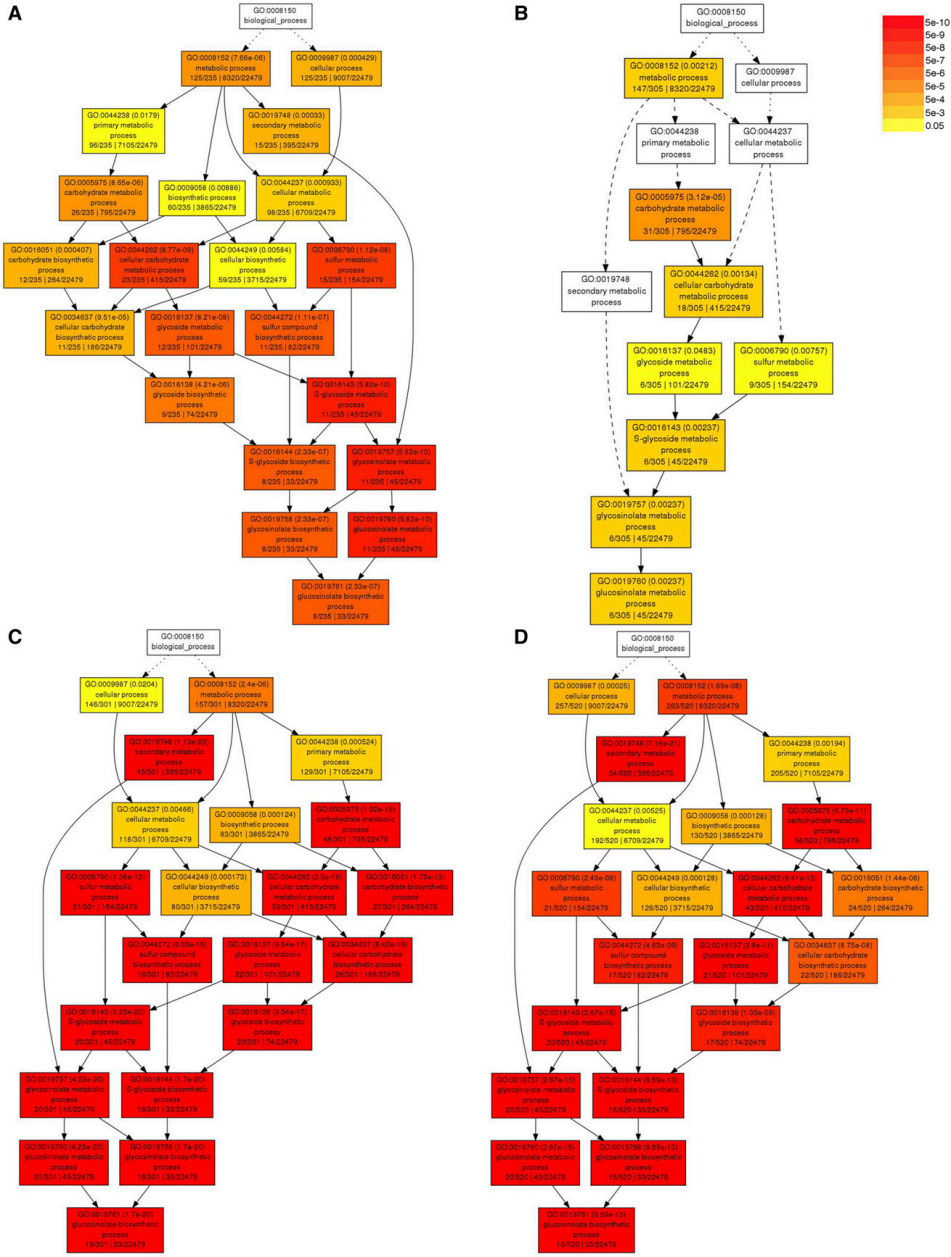
Hierarchical tree graphs of significantly enriched GO terms in glycoside metabolic pathways. A. Up-regulated genes in *ult1-3* 4D vegetative apices. B. Up-regulated genes in *ult1-3* IFM apices. C. Down-regulated genes in *35S:ULT1* IFM apices. D. Down-regulated genes in *clf-2* IFM apices. Non-significant GO terms are shown in white boxes and significant GO terms in colored boxes, with the color scale indicating the FDR-adjusted *p*-values from yellow (*P* < 0.05) to dark red (*P* < 5e-10). Solid, dashed and dotted lines represent two, one and zero enriched terms at the ends connected by the line, respectively. The information inside each colored box indicates: GO term number, adjusted *p*-value, GO description, number of items in the query list that map to the GO term / total number of items in the background that map to the GO term, and total number of items in the query list / total number of items in the background.

Our GO analysis indicated that GO categories related to GSL metabolic processes are among the most significantly enriched of both ULT1- and CLF-regulated genes. We find that GSL metabolic pathway genes are up-regulated in *ult1-3* vegetative and inflorescence apices (Figure 4A, B) and are down-regulated in 35S:*ULT1* over-expression lines (Figure 4C). These data indicate that ULT1 represses GSL metabolic gene transcription. Conversely, genes in this GO category are down-regulated in *clf-2* inflorescences (Figure 4D), showing that CLF induces their transcription. We investigated the extent to which ULT1 and CLF regulate glycoside metabolic gene transcription in greater detail by examining the relative expression levels of the pathways of genes involved in GSL biosynthesis, breakdown and transcriptional regulation.

**Figure 5:**
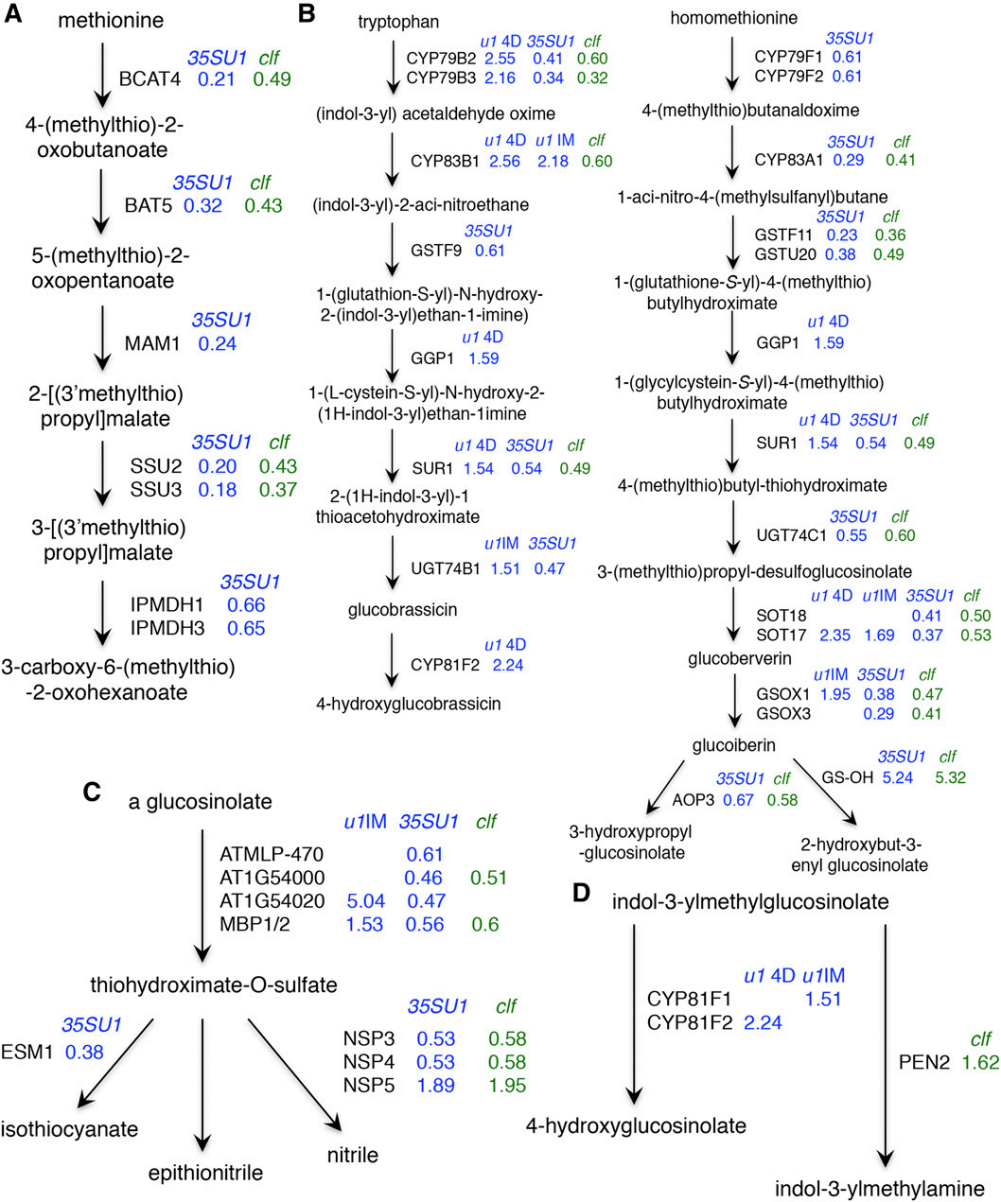
Glucosinolate pathway regulation by ULT1 and CLF. A. Enzymatic pathway for methionine side chain elongation. B. Enzymatic pathway for indolic GSL biosynthesis from tryptophan and alipathic GSL biosynthesis from homomethionine. C. Enzymatic pathway for GSL breakdown in response to tissue damage. D. Enzymatic pathway for GSL breakdown in intact tissue. Black arrows between compounds represent enzymatic steps catalyzed by the associated enzymes. Only enzymatic steps that involve ULT1- or CLF-regulated genes are shown. Enzyme-encoding genes regulated by ULT1 (blue) or CLF (green) are indicated along with the logFC in mRNA expression level, as detected by microarray analysis, in the given background. Pathways were drawn after biocyc.org and (Sønderby *et al.* 2010). *u1* = *ult1-3*, *35SU1* = 35S:*ULT1*, 4D = 4-day-old vegetative apices, IM = inflorescence meristem apices.

Most Arabidopsis GSLs are synthesized from either methionine or tryptophan (Sønderby *et al.* 2010). Before entering the main biosynthesis pathway, methionine undergoes side chain elongation, and genes encoding enzymes at multiple steps in this process are regulated by ULT1 or by both ULT1 and CLF (Figure 5A). The end products of the process are homomethionine and other chain-elongated derivatives, which then undergo biosynthesis of the core GSL structure. Again, genes encoding enzymes at most steps in this alipathic biosynthesis pathway, including the secondary modifications that create GSL structural diversity, are regulated by ULT1 or by both ULT1 and CLF (Figure 5B). The genes are repressed by ULT1 and/or induced by CLF, with the exception of the *GS-OH* gene, which functions in a later step of the pathway and is induced by both ULT1 and CLF. Indolic GSLs are synthesized from tryptophan, and similarly ULT1 and CLF regulate genes encoding nearly every step in this pathway, with the exception of *SOT16*, which functions in the step between *UGT74B1* and *CYP81F2* (Figure 5B).

Genes in the GSL breakdown pathways are likewise regulated by ULT1 and CLF. In response to tissue damage, GSLs are hydrolyzed by myrosinases and then converted into bioactive compounds through the activity of specifier proteins (Wittstock and Burow 2010). Several genes encoding myrosinase-associated proteins (*AtMLP-470*, *At1g54000* and *At1g54020*) are repressed by ULT1 and/or induced by CLF, along with two *MYROSINASE-BINDING PROTEIN* (*MBP1* and *MBP2*) genes (Figure 5C). In addition, ULT1 and CLF can oppositely regulate the expression of three of the five *NITRILE SPECIFIER PROTEIN* (*NSP*) genes. Indolic GSLs can also undergo breakdown in intact tissues via pathways involving an atypical myrosinase, PENETRATION2 (PEN2), or a set of cytochrome P450 (CYP) mono-oxygenases (Stahl *et al.* 2016). Our data indicate that *PEN2* expression is up-regulated in *clf-2* IFM apices, whereas *CYP81F2* is up-regulated in *ult1-3* vegetative and *CYP81F1* in *ult1-3* IFM apices (Figure 5D).

Finally, a handful of MYB and MYC transcription factors are known to regulate GSL metabolic processes, and several are downstream targets of ULT1 and/or CLF. The *MYB51* gene, a central regulator of indolic GSL biosynthesis in shoots upon salicylic acid and ethylene signaling (Frerigmann and Gigolashvili 2014), is repressed by ULT1 in 4D vegetative apices (Table S2). *MYB28* induces alipathic GSL biosynthesis (Gigolashvili *et al.* 2007) and is induced by CLF in reproductive apices (Table S5). Lastly, the *MYC3* gene is induced both by ULT1 and by CLF in reproductive apices (Tables S3, S5). MYC3 acts redundantly with MYC2 and MYC4 to directly activate GSL biosynthesis genes and physically associates with all known GSL-regulatory MYB proteins, including MYB28 and MYB51 (Schweizer *et al.* 2013).

Our data reveal that a total of 42 genes involved in GSL biosynthesis, breakdown or regulation are under the control of ULT1 and CLF during the plant life cycle. Among these, 25 (60%) can be regulated by both ULT1 and CLF, 15 by ULT1 alone, and only *PEN2* and *MYB28* by CLF alone. The observation that GSL pathway genes are up-regulated in *ult1-3* plants and/or down-regulated in 35S:*ULT1* and *clf-2* plants confirms that ULT1 acts as a repressor of GSL biosynthesis and breakdown whereas CLF functions as an inducer. We therefore conclude that the majority of genes regulating glycoside metabolic processes are oppositely regulated by the trxG factor ULT1 and the PcG factor CLF.

### Validation of microarray data

**Figure 6:**
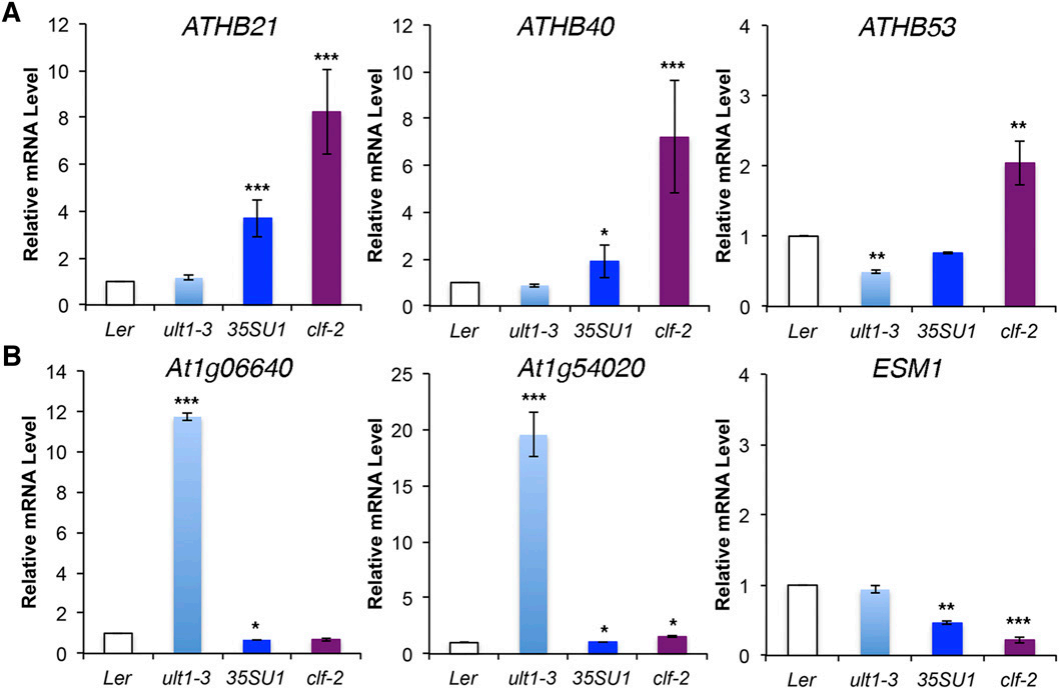
Validation of selected DEGs from the microarray data using RT-qPCR. A. Relative mRNA levels of the stress-responsive ULT1 and CLF target genes *ATHB21*, *ATHB40* and *ATHB53*. B. Relative mRNA levels of three ULT1 and CLF target genes in the glycoside metabolic pathway, *At1g06640*, *At1g54020* and *ESM1*. Expression levels (mean ± SD) were normalized to *TUB2* and expressed as a ratio to the level in wild-type plants. Asterisks indicate a significant difference from the wild-type mean (* = *P* < 0.05; ** = *P* < 0.01, *** =*P* < 0.001) using two-tailed Student’s *t*-test.

To validate the microarray results, we examined the mRNA levels of selected ULT1 and CLF target genes using reverse transcription-quantitative polymerase chain reaction (RT-qPCR). We chose genes from two categories of differentially expressed genes, abiotic stress response genes and glycoside metabolic genes, and quantified their expression levels in IFM apex tissue from wild-type L*er*, *ult1-3*, 35S:*ULT1* and *clf-2* plants grown under the same experimental conditions used for the microarray analysis. Overall, the quantitative gene expression results (Figure 6) correlated well with the trend of regulation observed in the microarray experiment.

*ATHB21*, *ATHB40* and *ATHB53* encode members of the HD-Zip class I family of transcription factors (Henriksson *et al.* 2005). These genes comprise the δ sub-class of HD-Zip I genes and are induced by application of abscisic acid (ABA) as well as salt, treatments that are associated with drought stress (Henriksson *et al.* 2005). We determined that the relative mRNA levels of the *ATHB21* and *ATHB40* genes were significantly elevated in 35S:*ULT1* and *clf-2* IFM apices, while *ATHB53* mRNA levels were significantly reduced in *ult1-3* and elevated in *clf-2* IFM apices (Figure 6A). Our results indicate that these genes are induced by ULT1 and repressed by CLF activity, consistent with ULT1 and CLF potentially playing antagonistic roles in response to abiotic stresses such as water deprivation.

We additionally tested three genes in glycoside metabolic pathways. The *At1g06640* gene encodes a 2-oxoglutarate-dependent dioxygenase that functions in methionine-derived GSL biosynthesis. The *At1g54020* gene encodes an S-glycosidase myrosinase-associated protein, and *EPITHIOSPECIFIER MODIFIER 1* (*ESM1*) encodes a GDSL-like carboxylic ester hydrolase; both of these proteins are involved in GSL breakdown (Zhang *et al.* 2006) (Figure 5C). We found that the expression levels of all three genes were significantly reduced in 35S:*ULT1* and *clf-2* IFM apices, and *At1g06640* and *At1g54020* expression was strongly up-regulated in *ult1-3* IFM apices (Figure 6B). We did not detect elevated *ESM1* expression in *ult1-3* IFM apices despite it being by far the most highly up-regulated gene in the *ult1-3* IFM apex microarray dataset (Table S3), suggesting the strength of that particular signal was an artifact. Nonetheless, our RT-qPCR results confirm that ULT1 and CLF play antagonistic roles in regulating glycoside metabolism, with ULT1 repressing and CLF inducing genes involved in the formation of GSLs and their active breakdown products during plant defense responses.

## DISCUSSION

trxG factors are epigenetic regulators that mediate the large-scale establishment and maintenance of active gene expression states. The SAND domain protein ULT1 has been characterized as a trxG factor based on functional criteria, including its ability to repress PcG mutant phenotypes and to associate with the trxG protein ATX1 (Carles and Fletcher 2009, Pu *et al.* 2013). However, the ULT1 protein lacks enzymatic activity, and its expression domain is restricted to meristematic and developing organ tissues (Carles *et al.* 2005), unlike other trxG factors such as the H3K4 methyltransferases ATXR3 and ATX1-ATX5, which are broadly expressed (Saleh *et al.* 2008, Berr *et al.* 2010, Guo *et al.* 2010, Chen *et al.* 2017). These observations suggest that ULT1 might function in a more limited set of molecular pathways than the H3K4 methyltransferase genes. Thus our rationale for performing a genome-wide transcriptome analysis was to determine whether ULT1 regulates a broad spectrum of plant pathways and processes like other known trxG factors or is restricted to those defined by its characterized developmental phenotypes (Fletcher 2001, Monfared *et al.* 2013). Because of ULT1’s restricted expression pattern, we preferentially sampled aerial meristematic and young organ tissues to enrich for *ULT1*-expressing cells within the vegetative or reproductive stage plants.

Our microarray analysis revealed that 723 genes, or only 2.6% of the total protein-encoding genes in the Arabidopsis genome, are mis-expressed in *ult1* vegetative and/or reproductive apices. This is a smaller proportion than found in a previous study, in which 9.3% of genes were mis-regulated in 7 DAG *ult1-3* seedlings and 8.1% in 15 DAG *ult1-3* seedlings (Pu *et al.* 2013). The difference is likely due to the use of whole-seedling tissues, short-day conditions and Agilent GeneChips in those experiments, all of which could expand the total repertoire of ULT1-regulated genes. The 2.6% value is also a smaller proportion than reported for most other plant trxG factors. For example, *ATXR3/SDG2*, which encodes the major H3K4 tri-methyltransferase in Arabidopsis, regulates the expression of approximately 2400 genes in 12 DAG seedlings (Guo *et al.* 2010). Another H3K4 tri-methyltransferase, ATX1, regulates ∼900 genes in four-week-old plants (Saleh *et al.* 2008) and ∼1640 genes at bolting (Alvarez-Venegas *et al.* 2006), whereas the H3K4 tri-methyltransferases ATX3, ATX4 and ATX5 redundantly regulate ∼1950 genes in three-week-old plants (Chen *et al.* 2017). In contrast, only 80 genes are regulated by the H3K4 di-methyltransferase ATX2 in four-week-old plants, and 58% of these gene targets are not shared with ATX1 (Saleh *et al.* 2008). Overall, little overlap exists between the transcriptomes of the ATX1, ATX2 and ATXR3 trxG proteins (Saleh *et al.* 2008, Guo *et al.* 2010), or among those of the other characterized trxG and PcG factors (Pu and Sung 2015). However, these various transcriptomics studies were performed using a variety of alleles, developmental stages and growth conditions, which are likely to amplify the differences between them and make direct comparisons of somewhat limited utility.

Although trxG factors are associated with the deposition of histone marks that promote active transcription states, ULT1 as well as the other trxG factors studied to date can function as positive as well as negative regulators of gene expression. More induced than repressed genes are found among the 80 ATX2 target loci, as 53 genes are down-regulated and 27 are up-regulated in *atx2* plants (Saleh *et al.* 2008). However, among the ATXR3, ATX1 and ATX3/4/5 target genes, the ratio of induced to repressed loci is approximately one to one (Alvarez-Venegas *et al.* 2006, Guo *et al.* 2010, Chen *et al.* 2017). We observe that among the 723 total ULT1 target genes, 187 are induced and 536 (74%) are repressed by ULT1. It may be that ULT1 indirectly represses the expression of many of these genes, or alternatively these findings may reflect that ULT1 can, in some situations, play a direct role in epigenetic gene silencing (Xu *et al.* 2018).

### Potential novel roles for ULT1 in regulating developmental processes

Analysis of the GO distribution networks of DEGs in our datasets indicated that genes associated with the GO term *developmental process* are over-represented in both *ult1-3* vegetative and IFM apices as well as 35S:*ULT1* IFM apices. This result is consistent with known roles for ULT1 in regulating shoot and floral meristem activity (Fletcher 2001, Carles *et al.* 2005), floral meristem termination (Carles and Fletcher 2009) and gynoecium patterning (Monfared *et al.* 2013, Pires *et al.* 2014). Yet the main ULT1 target genes in these three pathways – *WUSCHEL*, *AGAMOUS* and *SPATULA* – were not among the DEGs in our datasets. This is unsurprising because all of those genes have altered expression domains in *ult1* mutants rather than significantly different mRNA transcription levels. Therefore we do not expect that all of the developmentally relevant targets of ULT1 will have been identified through this transcriptomics study. Nonetheless, this approach has revealed potential functions for ULT1 in developmental processes with which it has not been previously associated.

Within the *developmental process* GO category for *ult1-3* 4 DAG up-regulated genes, genes involved in post-embryonic development and specifically root development are significantly enriched (Figure S2). Several ULT1-repressed TF genes in 4 DAG vegetative apices (Table 1) are involved in regulating root development. *LBD16* and *MYB77* both function in lateral root development in response to auxin (Shin *et al.* 2007, Lee *et al.* 2015), whereas *ERF109* acts during lateral root formation to integrate the auxin and jasmonic acid signaling pathways (Cai *et al.* 2014). *WRKY23* likewise functions in auxin-mediated root development (Prat *et al.* 2018), as well as in plant defense responses with which WRKY TFs are more typically associated (Eulgem and Somssich 2007). These observations suggest a potential role for ULT1 in repressing root developmental processes in the aerial tissues of seedlings.

In *ult1-3* IFM apices, genes associated with the GO terms *reproductive development* and *organ senescence* are up-regulated (Figure S4). Conversely genes associated with reproductive development are down-regulated in 35S:*ULT1* IFM apices (Figure S5), indicating that ULT1 may have as yet uncharacterized functions in repressing some aspects of flower development. In addition, ULT1 represses a number of TF genes in reproductive apices that regulate root and/or seedling growth. These include four members of the CCAAT gene family (Table 1), which encode subunits of the NF-Y transcription factor complex. This complex is involved in root growth and branching (Sorin *et al.* 2014) and the floral transition (Wenkel *et al.* 2006), as well as in abiotic stress responses (Leyva-Gonzalez *et al.* 2012). Other TF genes repressed by ULT1 in IFM apices affect processes such as photomorphogenesis (*BBX31*), leaf petiole growth (*ANAC47/SHYG*), and leaf anthocyanin production (*MYB90/PAP2*). These data are consistent with ULT1 acting to suppress some facets of vegetative development during the reproductive phase. Overall our study of ULT1-regulated target genes is consistent with a role for ULT1 in controlling developmental processes in a tissue- and/or stage-specific fashion.

### Role for ULT1 in regulating abiotic and biotic stress responses

Our microarray study demonstrates that ULT1 affects the transcription of genes in physiological and metabolic pathways as well as those in developmental pathways. The *response to stimulus* GO category is over-represented among the DEGs in *ult1-3* vegetative apices, *ult1-3* IFM apices and 35S:*ULT1* IFM apices, encompassing endogenous hormone responses as well as abiotic and biotic stress responses (Figure 2). Furthermore, nearly all the ULT1-regulated TF genes (Table 1), outside of those such as *FLC* and *CRC* that function in development, are involved in abiotic and/or biotic stress responses (Mittler *et al.* 2006, Moffat *et al.* 2012, Kim *et al.* 2015, Zhao *et al.* 2017).

Due to their sessile nature, plants can face exposure to a variety of abiotic stresses during their lifetimes from changing environmental conditions. The contribution of trxG and PcG factors to abiotic stress responses is not well understood. However, enrichment of H3K4me3 marks at some stress-responsive genes has been associated with a proposed cellular memory system induced by environmental stresses such as drought and heat (Ding *et al.* 2012a, Lämke *et al.* 2016), the former involving the trxG factor ATX1 (Ding *et al.* 2011b). Our data show that genes associated with responses to oxidative stress, water deprivation and cold are among the significantly DEGs in *ult1-3* and 35S:*ULT1* IFM apices (Figure 2, Table S3, S4). These DEGs include the *ATHB21*, *ATHB40* and *ATHB53* HD-Zip I TF genes that are induced by drought stress (Henriksson *et al.* 2005) as well as four CCAAT family TF genes that promote drought and cold stress responses (Zhao *et al.* 2017). Currently the only described involvement of ULT1 in abiotic stress responses is the demonstration that the *ult1-3* allele can attenuate the salt tolerance phenotype of plants with reduced *EMF1* activity (Pu *et al.* 2013). Whether the observed changes in abiotic stress-responsive gene transcript levels in *ult1-3* plants are sufficient to confer quantifiable phenotypes remains an open question. Further molecular and physiological analysis will be required to determine the role of ULT1 in these fundamental biological processes.

Plants are also under constant threat from animals, insects and various pathogens, and we find that ULT1 regulates many classes of genes involved in biotic stress pathways. GO category terms related to *innate immune response*, *response to wounding* and *response to fungus* are significantly enriched among ULT1-repressed genes in vegetative apices; genes in the latter two categories are also repressed by ULT1 in reproductive apices (Figure 2). Also within the *metabolic process* GO category, ULT1 regulates genes in several pathways that mediate plant defense responses: oxidation/reduction metabolic pathways and glycoside metabolic pathways. Redox pathways play important roles in plant immunity, as the production of reactive oxygen species occurs rapidly in response to pathogen attack and induces immune functions such as the hypersensitive response (Frederickson Matika and Loake 2014). Glycosides such as GSLs are typically activated upon wounding and form secondary metabolites that are toxic to micro-organisms, nematodes and insects, thereby contributing to plant-herbivore and plant-pathogen defense responses (Wittstock and Burow 2010, Bednarek 2012). Consistently, the ULT1-regulated DEGs in the GSL pathways (Figure 5) strongly overlap with those in the *response to wounding* GO category. These results suggest a heretofore unknown role for ULT1 in regulating induced as well as innate plant defense responses, particularly during the vegetative phase.

Although histone marks including H3K4me3 and H3K9ac are associated with some immune responses such as systemic acquired resistance (Jaskiewicz *et al.* 2011), specific functions for trxG factors (or PcG factors) in biotic stress responses remain undiscovered. One study has implicated the histone acetyltransferase HAC1 in environmental stress-induced bacterial resistance and pattern-triggered immunity priming (Singh *et al.* 2014). Because *HAC1* expression is repressed by ULT1 in both vegetative and IFM apices (Table S2, S3), it is possible that *HAC1* regulation by ULT1 may contribute to plant defense responses. Our data also reveal a potential role for CLF in plant defense responses. Analysis of GO distribution networks of DEGs from *clf-2* IFM apices demonstrated that biotic stress genes were significantly enriched among both CLF-induced and -repressed genes, and some of the most highly up-regulated genes in *clf-2* IFM apices are the pathogenesis-related genes *PATHOGENESIS-RELATED PROTEIN1* (*PR1*), *PR2*, and *PR5* (Table S5). Also, like ULT1, CLF regulates multiple components of the plant immunity-related oxidation/reduction metabolic pathways and glycoside metabolic pathways (Figure 2, 5). Future studies to determine the contributions of ULT1 and CLF, as well as other trxG and PcG factors, to plant defense responses may provide new insights into the molecular mechanisms that regulate innate and induced immune response pathways and that coordinate these responses with the appropriate alterations in plant growth.

The *ULT1* trxG gene and the *CLF* PcG gene have a clear genetic and morphological association. ULT1 was originally defined as a trxG factor based on the ability of *ult1* null alleles to suppress the *clf* developmental phenotypes, and 35S:*ULT1* plants appear indistinguishable from *clf* plants (Carles and Fletcher 2009). Our transcriptome data reveal that nearly a third of DEGs in *ult1-3* IFM apices (Figure 1C) and three quarters of DEGs in 35S:*ULT1* IFM apices (Figure 1D) are also regulated by CLF, providing a molecular basis for the overlap in developmental phenotypes observed. However, ULT1 has a smaller effect on CLF-mediated gene regulation, likely because the former controls the expression of a smaller suite of genes. Interestingly, our results indicate that ULT1 and CLF antagonistically affect molecular pathways beyond development, notably the glucosinolate metabolic pathways in which ULT1 represses and CLF induces the expression of genes involved in GSL biosynthesis and in GSL activation (Figure 5). Finally, an RNA-seq study showed that CLF-mediated gene transcriptional repression is highly tissue-specific (Liu *et al.* 2016), perhaps because CLF associates with different PcG partner proteins to regulate distinct developmental programs (Wang *et al.* 2016). Although ULT1 has not yet been shown to interact with trxG factors other than ATX1, the fact that ULT1 associates with transcription factors like UIF1 and KAN1/2 that have distinct expression patterns (Kerstetter *et al.* 2001, Moreau *et al.* 2016) suggests that ULT1 may likewise function in multiple protein complexes to carry out stage- and tissue-specific gene regulation of diverse processes during the Arabidopsis life cycle.
